# Temporal and topological properties of dynamic networks reflect disability in patients with neuromyelitis optica spectrum disorders

**DOI:** 10.1038/s41598-024-54518-7

**Published:** 2024-02-20

**Authors:** Yao Wang, Ziwei Yang, Xiumei Zheng, Xiao Liang, Jin Chen, Ting He, Yanyan Zhu, Lin Wu, Muhua Huang, Ningnannan Zhang, Fuqing Zhou

**Affiliations:** 1 Department of Radiology, The First Affiliated Hospital, Jiangxi Medical College, Nanchang University, Nanchang, 330006 Jiangxi Province China; 2 Clinical Research Center for Medical Imaging in Jiangxi Province, Nanchang, 330006 Jiangxi Province China; 3 Department of Neurology, The First Affiliated Hospital of Nanchang University, Nanchang, 330006 Jiangxi Province China; 4 Department of Radiology, Pingxiang People’s Hospital, Pingxiang, 337055 Jiangxi Province China; 5 Department of Radiology and Tianjin Key Laboratory of Functional Imaging, Tianjin Medical University General Hospital, Tianjin, 300052 China

**Keywords:** Neurological disorders, Functional magnetic resonance imaging

## Abstract

Approximately 36% of patients with neuromyelitis optica spectrum disorders (NMOSD) suffer from severe visual and motor disability (blindness or light perception or unable to walk) with abnormalities of whole-brain functional networks. However, it remains unclear how whole-brain functional networks and their dynamic properties are related to clinical disability in patients with NMOSD. Our study recruited 30 NMOSD patients (37.70 ± 11.99 years) and 45 healthy controls (HC, 41.84 ± 11.23 years). The independent component analysis, sliding-window approach and graph theory analysis were used to explore the static strength, time-varying and topological properties of large-scale functional networks and their associations with disability in NMOSD. Compared to HC, NMOSD patients showed significant alterations in dynamic networks rather than static networks. Specifically, NMOSD patients showed increased occurrence (fractional occupancy; *P* < 0.001) and more dwell times of the low-connectivity state (*P* < 0.001) with fewer transitions (*P* = 0.028) between states than HC, and higher fractional occupancy, increased dwell times of the low-connectivity state and lower transitions were related to more severe disability. Moreover, NMOSD patients exhibited altered small-worldness, decreased degree centrality and reduced clustering coefficients of hub nodes in dynamic networks, related to clinical disability. NMOSD patients exhibited higher occurrence and more dwell time in low-connectivity states, along with fewer transitions between states and decreased topological organizations, revealing the disrupted communication and coordination among brain networks over time. Our findings could provide new perspective to help us better understand the neuropathological mechanism of the clinical disability in NMOSD.

## Introduction

Neuromyelitis optica spectrum disorder (NMOSD) is an immune-mediated inflammatory disease that mainly involves the optic nerve and spinal cord^1^, and approximately 36% of patients suffer from severe visual and motor disability (blindness or light perception or unable to walk) in China^2^. The pathophysiological mechanisms of disability are complex and not fully understood. Lesions and atrophy of the spinal cord^3^ or white matter (WM)^4^ and deep gray matter^5^ were only weakly or moderately associated with physical disability, indicating that other processes might play crucial roles in disability in patients with NMOSD.

Functional network refers to the complex network stems from functional interactions between brain regions. Considering the complexity of the neurobiological mechanisms underlying disability in NMOSD, it is crucial to investigate the involvement of multiple functional networks. Many studies have demonstrated functional network reorganizations in NMOSD^6–8^, and have revealed correlations between certain static functional networks and disability or motor. Specifically, increased static functional connectivity of the basal ganglia network^9^, and working memory network^10^ were correlated with lower disability score in NMOSD. Furthermore, higher connectivity of auditory network was related to better motor performance of the 9-hole peg test^11^ in patients with NMOSD. These static function connectivity focuses on the overall organization of brain networks, characterizing the stable and long-term connectivity patterns between brain regions in NMOSD. In addition, brain networks are also “dynamic”, exhibiting fluctuations and changes in brain oscillations and connectivity patterns over time during the scanning process^12^. Complementary with static connectivity, dynamic connectivity analysis captures the temporal fluctuations in brain connectivity patterns, revealing the instantaneous interactions between brain regions^13^. Many studies have suggested that quantifying time-varying functional connectivity may provide additional information on the fundamental properties of brain networks^14^. Recently, one study reported that increased dynamic connectivity in the precuneus could better explains depressive symptoms and cognitive impairment in NMOSD patients than static connectivity^15^.

The topology is a branch of mathematics that investigates the properties and relationships of space^16^. It provides a framework for understanding the fundamental structure and characteristics of various objects and systems. Topology is particularly crucial for comprehending the brain as a complex network. The brain consists of a intricate network of neurons and their connections forming a complex neural network^17^. By applying topology, particularly through graph theory analysis, the essential features like the small-world characteristics and hub regions of the brain network can be revealed^16^. These indicators play a significant role in characterizing the overall and local efficiency of the brain's information integration and processing. In graph theory, brain regions can be regarded as sets of nodes, and the functional network topology can be characterized by assessing the interaction strength and information processing efficiency between spatially distinct areas^18,19^. The graph theory analysis have been used to further reveal how NMOSD affects information processing and functional integration within structural and functional networks by examining connectivity patterns and network properties. For instant, reduced global and local efficiency and altered nodal properties in WM networks associated with disability and cognitive performance were observed in patients with NMOSD^20–22^. Another study investigated functional networks, and reported no significant difference in global topology between NMOSD and healthy controls (HC), including the small-world property^6^. The preservation of the small-world property in the functional network could highlight the adaptability and flexibility of the brain's functional organization in NMOSD. Nevertheless, the topological properties of static and dynamic networks in NMOSD patients have rarely been studied.

Therefore, our study hypothesizes that connectivity strength and topological features decreases in both static and dynamic functional networks in NMOSD and that these alterations are associated with clinical disability. To evaluate these hypotheses, we constructed brain networks, and static functional network connectivity (sFNC) analyses were performed to assess the mean connectivity strength and dynamic functional network connectivity (dFNC) to investigate the time-varying characteristics of these networks. Finally, graph theoretical analysis was applied to evaluate the topological properties of sFNC and dFNC. Our study aims to explore stable network architecture and dynamic changes occurring in the functional networks and lead to a deeper understanding of the underlying mechanisms of disability in NMOSD.

## Methods and materials

### Subjects

For this prospective study, all participants signed informed consent forms, and our study was approved by the Ethics Committee of the First Affiliated Hospital of Nanchang University (Granted No.: 2021-4-074) and carried out in accordance with Declaration of Helsinki. The inclusion criteria were defined as follows: patients were 18–65 years old, met the 2015 revised diagnostic criteria for NMOSD patients without traumatic brain injury and neuropsychiatric history, and demographic information was provided. All patients completed Expanded Disability Status Scale (EDSS) tests within 2 h of the magnetic resonance imaging (MRI) scan. The exclusion criteria were defined as follows: a history of head injury or other neuropsychiatric diseases, poor image quality (e.g. artifacts, distortions or incomplete brain coverage), incomplete clinical information or obvious head motion (head motion ≥ 3° and ≥ 3 mm). A total of 30 patients with NMOSD and 45 matched HC were included, and data collected between January 2017 and August 2022 were analyzed.

### MRI acquisition

All subjects were scanned with a Trio 3.0 T MRI scanner (Siemens, Munich, Germany) following a standardized protocol: (1) resting-state functional MRI (rs-fMRI) acquisition [repetition time (TR)/echo time (TE) = 2000/30 ms, flip angle (FA) = 90°, matrix = 64 × 64, field of view (FOV) = 210 × 210 mm, and 240 time points]; (2) high-resolution 3D T1-weighted image acquisition (TR/TE = 1900/2.26 ms, matrix = 240 × 256, FOV = 215 × 230 mm, and 176 sagittal slices); (3) T2-fluid attenuated inversion recovery (FLAIR) sequences (TR/TE = 7000/77 ms, inversion time = 2500, FA = 120°, slice thickness: 2 mm, and 50 slices). During rs-fMRI acquisition, subjects were asked to close their eyes, relax and remain awake with minimal specific thinking.

### Brain volume measures

We performed preprocessing on 3D T1-weighted images using CAT12 (http://www.neuro.uni-jena.de/cat/). The T1-weighted images were segmented into gray matter and white matter by default settings and calculated the gray matter volume (GMV), white matter volume (WMV) and total intracranial volume (TIV). The brain parenchymal fraction (BPF) is defined as the ratio of brain tissue (GMV + WMV) to TIV. Additionally, the lesion volumes (LV) were calculated utilizing the Lesion Segmentation Toolbox (LST; https://www.statistical-modelling.de/lst.html) based on T2-FLAIR sequences of NMOSD. Using the lesion prediction algorithm with recommended threshold kappa (k = 0.5), the binary lesion maps were obtained to calculate LV for individual NMOSD patients.

### Functional MRI preprocessing

Functional MRI data were preprocessed using the Resting-State fMRI Data Analysis Toolkit (RESTplus) v1.25 based on SPM12 software. The preprocessing pipeline included the following steps: (1) The data were converted from DICOM to NIFTI format. (2) The first 10 volumes were removed. (3) Slice timing operations were performed. (4) Motion correction operations were performed. Subjects with a maximum translation > 3 mm or rotation > 3° were excluded. In addition, according to the work of Jenkinson et al.^23^, the mean framewise displacement (FD) of each subject was calculated to reflect the specific value of head movement. (5) The images were normalized in the MNI space and resampled with 3 × 3 × 3 mm^3^ voxels. (6) The images were spatially smoothed with a 6 mm full width at half maximum Gaussian kernel.

### Independent component analysis

Spatial ICA (GIFT v3.0b, http://mialab.mrn.org/software/gift) was performed on the MATLAB platform based on the rs-fMRI data of all participants. According to the study conducted by Allen et al.^24^, principal component analysis and the Infomax algorithm were applied to obtain 75 independent components (ICs) based on special-subject time courses. The Infomax algorithm was iterated 100 times, and the process was repeated 5 times with ICASSO to estimate the reliability of the ICs. Forty-three ICs with peak activations in the gray matter were selected, and ICs with spatial overlap in the white matter, ventricles, and edge regions were excluded. Next, the time courses of the 43 ICs were postprocessed by detrending the linear, quadratic and cubic trends, regressing out 6 realignment parameters and their temporal derivatives, despiking, and bandpass filtering between 0.01 and 0.15 Hz using a 5th order Butterworth filter.

Resting-state networks (RSNs) refer to the functional connectivity patterns observed in the brain during a resting state, which characterized by synchronized activity among spatially distributed brain regions. Evaluating RSNs plays a crucial role in understanding the intrinsic functional organization of the brain and how different brain regions interact and communicate with each other in patients with NMOSD. The formation of RSNs in this study was guided by the work of Yeo et al.^25^, who identified seven brain networks. Considering the cerebellar network as distinct from the cortex networks, eight networks were thus determined in our study. To establish the correspondence between the 43 ICs derived from ICA and the RSNs, spatial regression analysis and visual inspection were conducted to identify the corresponding network components for further investigation.

### sFNC analysis

First, the postprocessed time courses of the sFNC were transformed to Fisher-z scores, and paired Pearson’s correlation coefficients were calculated. A static connectivity matrix with 903 pairs was obtained for each subject to evaluate the intra- and internetwork connectivity strength.

### dFNC analysis

Next, the sliding window method in the Temporal dFNC module of GIFT v3.0b software was used to assess the dFNC. The specific parameters were set as follows: the window width was set to 30 TRs (60 s), and the step size was set to 1 TR (2 s). The L1 regularization method (10 repetitions) was applied in the dFNC rather than sFNC to promote the sparsity and identify the most influential dynamic connectivity within the network. In contrast, the sFNC analysis aimed to capture the overall connectivity patterns without emphasizing individual connectivity strengths. For each subject, a total of 198 windows were obtained to reflect the time-varying functional connectivity among all ICs. The Fisher-Z transformation was applied to each correlation coefficient in the dFNC matrices, promoting normality and stabilization of variance. Subsequently, the confounders, including the mean FD, age and sex, were regressed out from the transformed dFNC windows. Then, the k-means algorithm was used to cluster the dFNC matrix for each window across all subjects and extract recurring dFNC patterns. The similarity across different windows was measured according to the Manhattan distance (150 iterations and 5 repetitions). The cluster centroids represented reoccurring “brain states” using K-means clustering. According to the elbow criterion^26^, the optimal number of cluster centroids was defined as the ratio of the within-cluster distance to the between-cluster distance.

Finally, we evaluated the state-related properties of the dFNC: (1) The Fractional occupancy refers to the percentage of overall time that each subject spent in a specific state; (2) the mean dwell time represents the average duration of time that each subject spent in each state; and (3) the number of transitions indicates the number of times that each subject transitioned between different states.

### Dynamic topological analysis

The topological properties of the sFNC and dFNC matrices were analyzed in GRETNA_2.0. We defined the 43 ICs as independent nodes and the pairwise correlation coefficients as edges. In order to explore the topological characteristics of the sFNC and dFNC in NMOSD, a series of sparse thresholds were employed to binarize the functional networks. Only the absolute values of the edges that exceeded the threshold were retained and defined as 1, while the rest smaller than the threshold were set to 0. Taking reference from previous studies^27^, we set the range of the sparse threshold between 0.10 and 0.38 (with an interval of 0.01). According to previous studies^28^, the area under the curve (AUC) of the network metrics was calculated for each sparse threshold.

For each sparse threshold, we calculated the global and nodal measures for the sFNC and dFNC across different windows. The global measures included the AUC of the small-world global metrics, such as the clustering coefficient (*aCp*), normalized clustering coefficient (*aGamma*), shortest path length (*aLp*), normalized characteristic path length (*aLambda*), and small-worldness (*aSigma*). The regional metrics included the nodal clustering coefficient, degree centrality and nodal efficiency. The brief descriptions of topological measures is shown in Table S1. For dynamic networks, the variance of the AUC values of the networks was calculated to reflect the variability in the network properties over time^29,30^.

### Statistical analysis

We performed Kolmogorov‒Smirnov tests to assess the normality; the mean ± standard deviation are reported for normally distributed data, and the median and interquartile range are reported for nonnormally distributed data. An analysis of covariance (ANCOVA) was used to compare the sFNC strength [*P* < 0.05, false discovery rate (FDR) corrected]. The independent samples t-test was used for demographic data and brain volume measures. The Mann‒Whitney U test (*P *< 0.05) was used to assess the state-related properties of dFNC and topological measures in two groups. The effect size of the Mann–Whitney U test is calculated using the rank-biserial correlation coefficient and is presented as the absolute value, which located 0–1. When the effect size is close to 1, it means more significant difference between the two groups. For graph theory-based metrics of the networks, no correction was applied to take the number of measures into account between two groups, but FDR correction (*P* < 0.05) for the nodal measures was performed due to the number of ICs between groups. Spearman rank correlation scores were calculated between significant measures and EDSS scores for patients with NMOSD (*P* < 0.05).

### Informed consent

All participants signed informed consent forms.

## Results

Table 1 summarizes the demographic and clinical information of NMOSD (N = 30) and HC (N = 45). Patients with NMOSD displayed a short disease duration (median: 1.80 years) and mild disability (median EDSS score: 1.25). Compared to HC, the NMOSD group showed white matter atrophy (*P* = 0.004), but there was no difference in gray matter (*P* = 0.068). Among patients with NMOSD, only 33% (N = 10) of patients had brain lesions with low lesion burden (median LV = 1.22 ml). The most common location for lesions was found in the brainstem (N = 5, 17%), followed by the area postrema (N = 4, 13%). Table S2 summarizes the detailed the image finding in NMOSD. There were no statistically significant differences in age, sex or mean FD (*P*: 0.080–0.195).

**Table 1:** Demographic and clinical information.

	NMOSD (N = 30)	HCs (N = 45)	T or Phi value	*P* value
Age (years)^a^	37.70 ± 11.99	41.84 ± 11.23	1.52	0.132
Sex (female/male)	27/3	33/12	0.20	0.080
Mean FD (mm)^a^	0.04 ± 0.03	0.05 ± 0.04	1.31	0.195
DD (years)^b^	1.80 (0.60–4.50)	/	/	/
TIV (ml)	1341.7 ± 108.98	1385.07 ± 106.30	1.71	0.091
GMV (ml)^a^	571.67 ± 39.20	593.47 ± 55.95	1.85	0.068
WMV (ml)^a^	475.88 ± 46.33	510.86 ± 52.42	2.96	0.004
BPF^a^	0.78 ± 0.04	0.79 ± 0.03	1.99	0.051
LV (ml)^b^	1.22 (0.69–1.82)	/	/	/
EDSS scores^b^	1.25 (1.00–1.63)	/	/	/

*NMOSD* neuromyelitis optica spectrum disorder, *HCs* healthy controls, *FD* frame-wise displacement, *DD* disease duration, *EDSS* Expanded Disability Status Scale, *GMV* gray matter volume, *WMV* white matter volume, *TIV* total intracranial volume, *BPF* brain parenchymal fraction, *LV*, lesion volumes.

^a^Indicates mean ± standard deviation.

^b^Indicates median (interquartile range).

### dFNC and sFNC analyses

**Figure 1:**
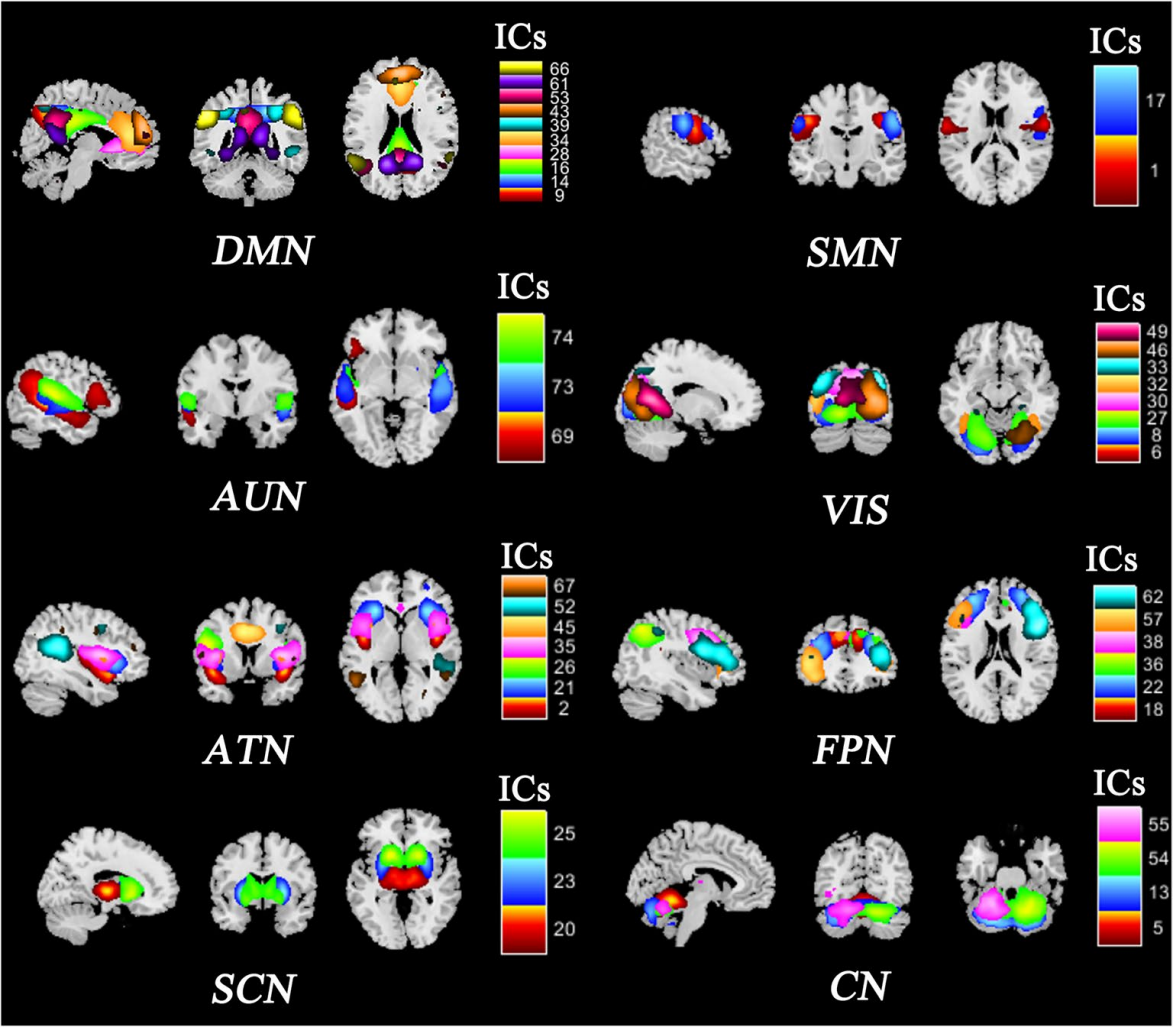
ICs and RSNs. *Note*: ICs, independent components; RSNs, resting-state networks; DMN: default mode network; SMN: sensorimotor network; ADN, auditory network; VIS, visual network; ATN, attention network; FPN, frontoparietal network; SCN, subcortical network; CN, cerebellar network.

Eight RSNs were defined at the whole-brain level: (1) the default mode network (DMN); (2) the sensorimotor network (SMN); (3) the auditory network (ADN); (4) the visual network (VIS); (5) the attention network (ATN); (6) the frontoparietal network (FPN); (7) the subcortical network (SCN); and (8) the cerebellar network (CN). The spatial maps and peak coordinate for the ICs and the corresponding RSNs are shown in Fig. 1 and Table S3.

### General state-related properties of the dFNC

**Figure 2:**
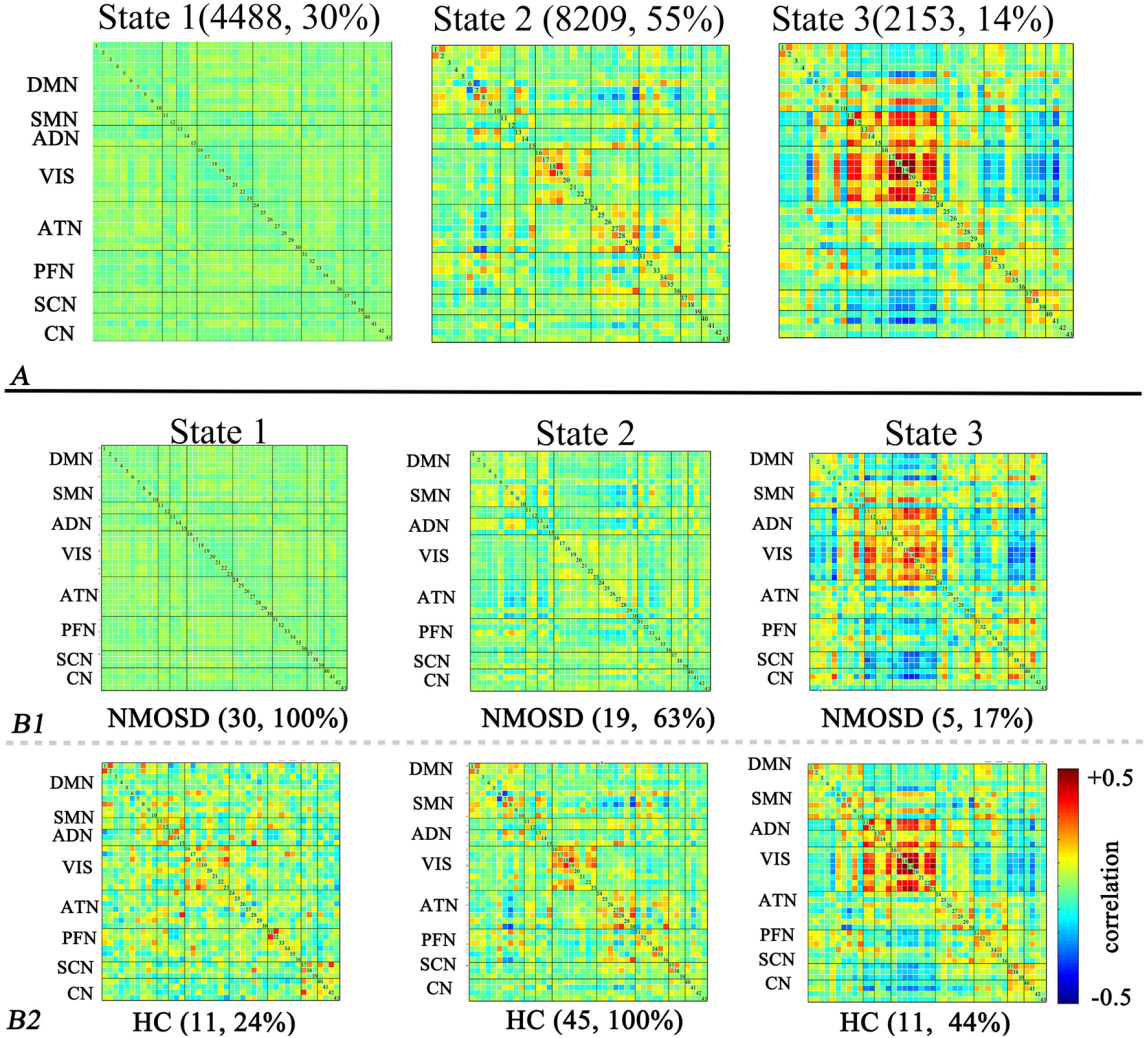
dFNC states for NMOSD patients and HC. *Note*: The optimal number of dFNC states and the proportion of each state for all subjects (**A**). The occurrence and proportion of specific states in the NMOSD (**B1**) and HC groups (**B2**). *Note*: NMOSD, neuromyelitis optica spectrum disorder; HC, healthy control; DMN: default mode network; SMN: sensorimotor network; ADN, auditory network; VIS, visual network; ATN, attention network; FPN, frontoparietal network; SCN, subcortical network; CN, cerebellar network.

After the K-means clustering analysis was performed, three dFNC states were defined for all windows (Fig. 2A): the lowly connected state (State 1), the moderately connected state (State 2) and the strongly connected state (State 3). State 1 occurred moderately frequently (30%) and characterized by the sparse connectivity within and between networks. State 2 occurred at the most frequency (55%) and was characterized by increased coupling within the DMN, VIS and ANT. State 3 also exhibits positive connectivity within VIS, and negative connections between VIS and FPN or SCN with a less frequency (14%). Figure 2B1,B2 display the different tendencies observed in the NMOSD patients and HC: in NMOSD, the proportion of individuals in a specific states is as follows: State 1 (100%), State 2 (63%), and State 3 (17%); the proportion of individuals in a specific states is as follows: State 1 (24%), State 2 (100%), and State 3 (44%) in HC.

### Between-group difference in the state-related properties of the dFNC

**Figure 3:**
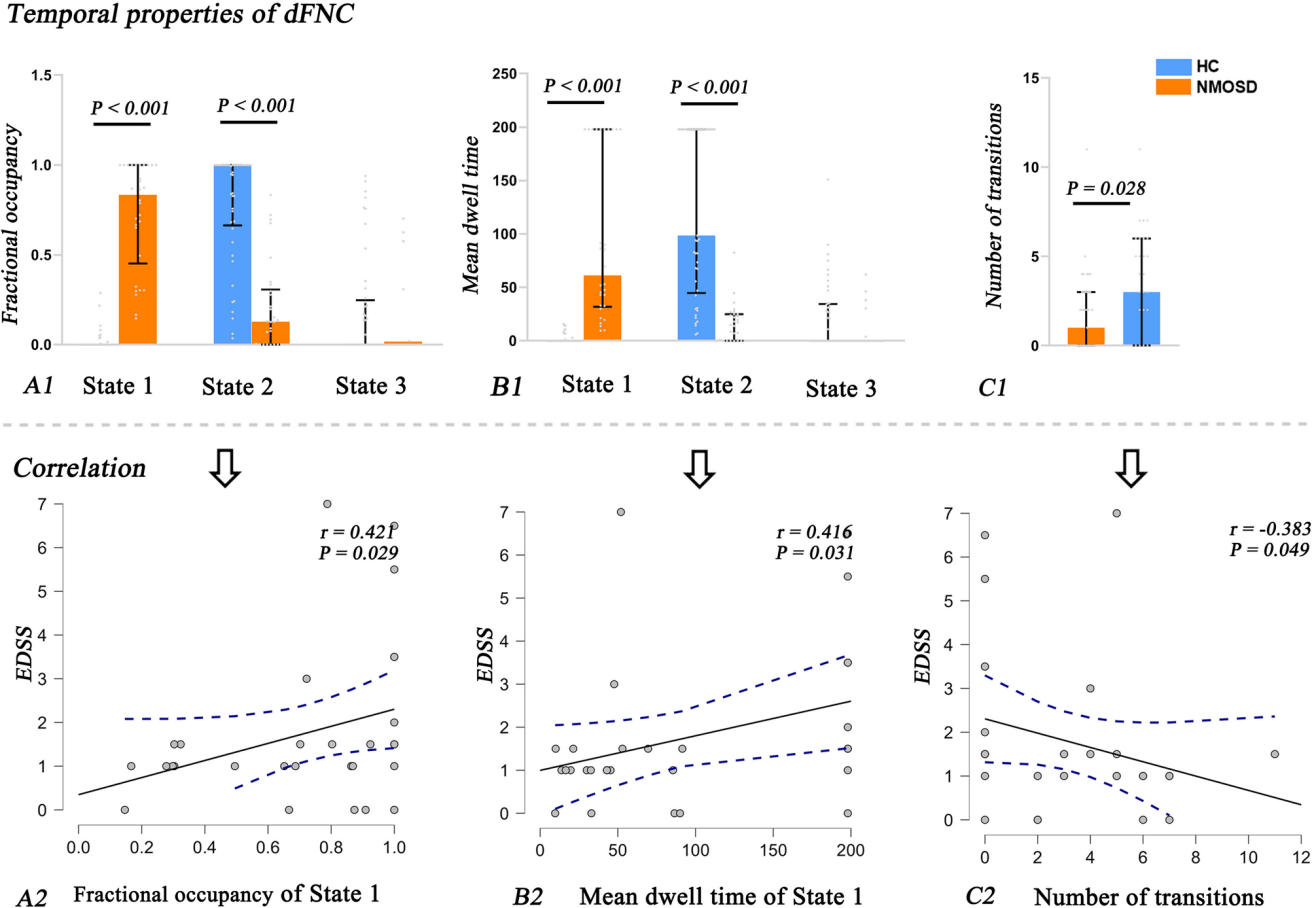
Significant differences in the fractional occupancy (**A1**), mean dwell time (**B1**) of each state and number of transitions (**C1**) between states in the NMOSD and HC groups. The correlation results between the fractional occupancy (**A2**), mean dwell time (**B2**) and number of transitions (**C2**) and the EDSS scores in patients with NMOSD. *Note*: NMOSD, neuromyelitis optica spectrum disorder; HC, healthy control. EDSS, Expanded Disability Status Scale.

We used the fractional occupancy, mean dwell time and number of transitions to characterize the state-related properties of the dFNC. The group-level results revealed that patients with NMOSD showed higher fractional occupancy in State 1 (uncorrelated *P* < 0.001) and longer dwell times in State 1 (uncorrelated *P* < 0.001), while HC tended to spend more time in State 2 (uncorrelated *P* < 0.001) (Fig. 3A1,B1). Regarding the number of transitions, patients with NMOSD showed fewer transitions between different states than HC (uncorrelated *P* = 0.028) (Fig. 3C1).

In patients with NMOSD, the higher fractional occupancy (*r* = 0.421, *P* = 0.029) and increased dwell time (*r* = 0.461, *P* = 0.031) in State 1 were correlated with higher EDSS scores (Fig. 3A2,B2). Moreover, the reduced number of transitions was associated with higher EDSS scores (*r* =  − 0.383, *P* = 0.049) in patients with NMOSD (Fig. 3C2).

### sFNC analysis

sFNC was used to assess the mean connectivity strength during fMRI scanning. No significant difference in the sFNC matrices was observed between patients with NMOSD and HC.

### Dynamic topological analysis

#### Global properties of dFNC

**Figure 4:**
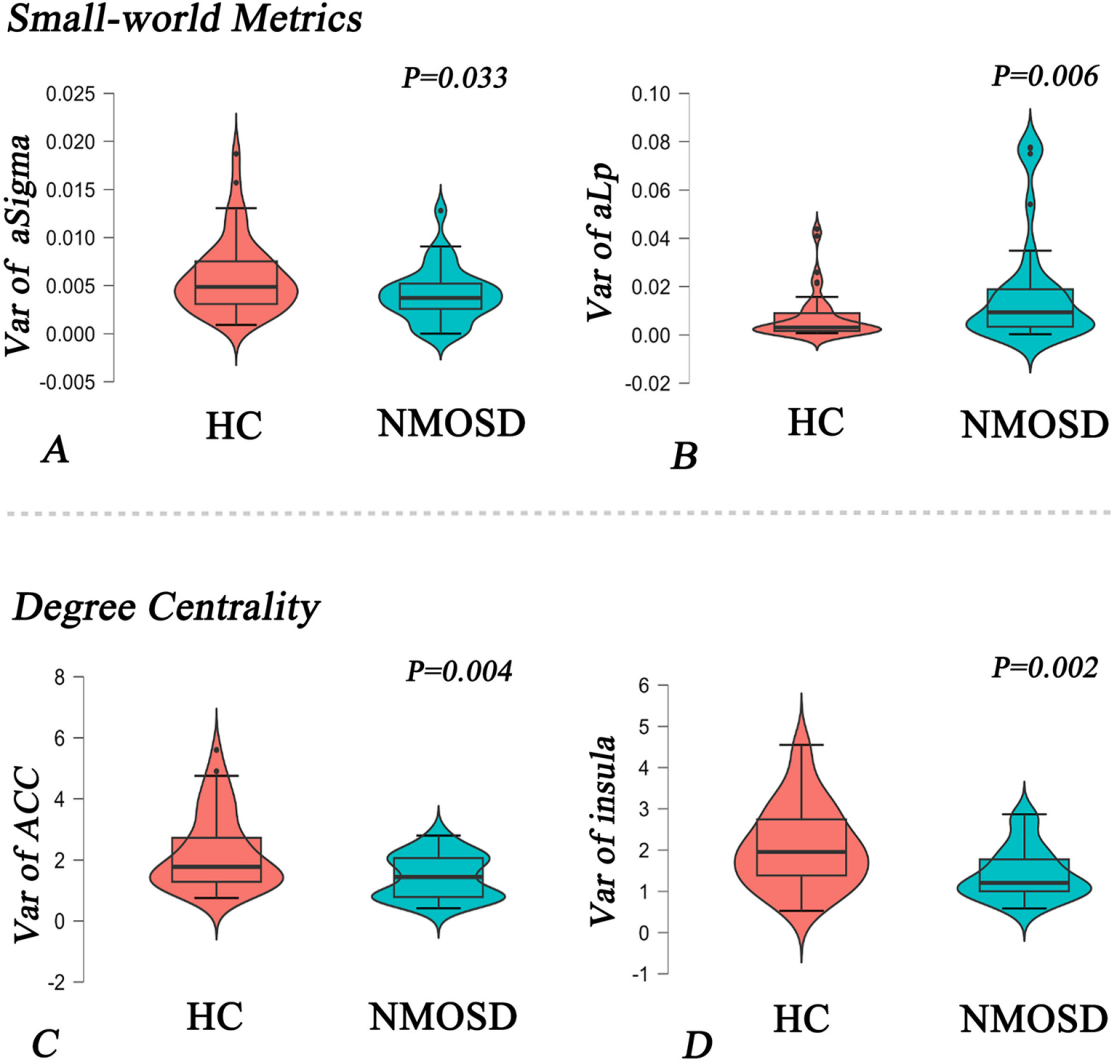
NMOSD group exhibited decreased variance for *aSigma* (**A**), increased variance for *aLp* (**B**), increased variance for small-world metrics and reduced variance for nodal degree centrality (**C**, **D**). *Note*: NMOSD, neuromyelitis optica spectrum disorder; HC, healthy control; Var, variance; ACC, bilateral anterior cingulate cortex.

In terms of the global topological properties of brain networks, patients with NMOSD showed decreased variance for *aSigma* (uncorrelated *P* = 0.033) and *aLp* (uncorrelated *P* = 0.006) in small-world features (Fig. 4A,B). However, significant group-level difference between NMOSD patients and HC was not observed for other small-world metrics or the global efficiency (*aCp*, *P* = 0.671; *aGamma*,* P* = 0.106; *aLambda*,* P* = 0.179).

#### Nodal properties of dFNC

**Figure 5:**
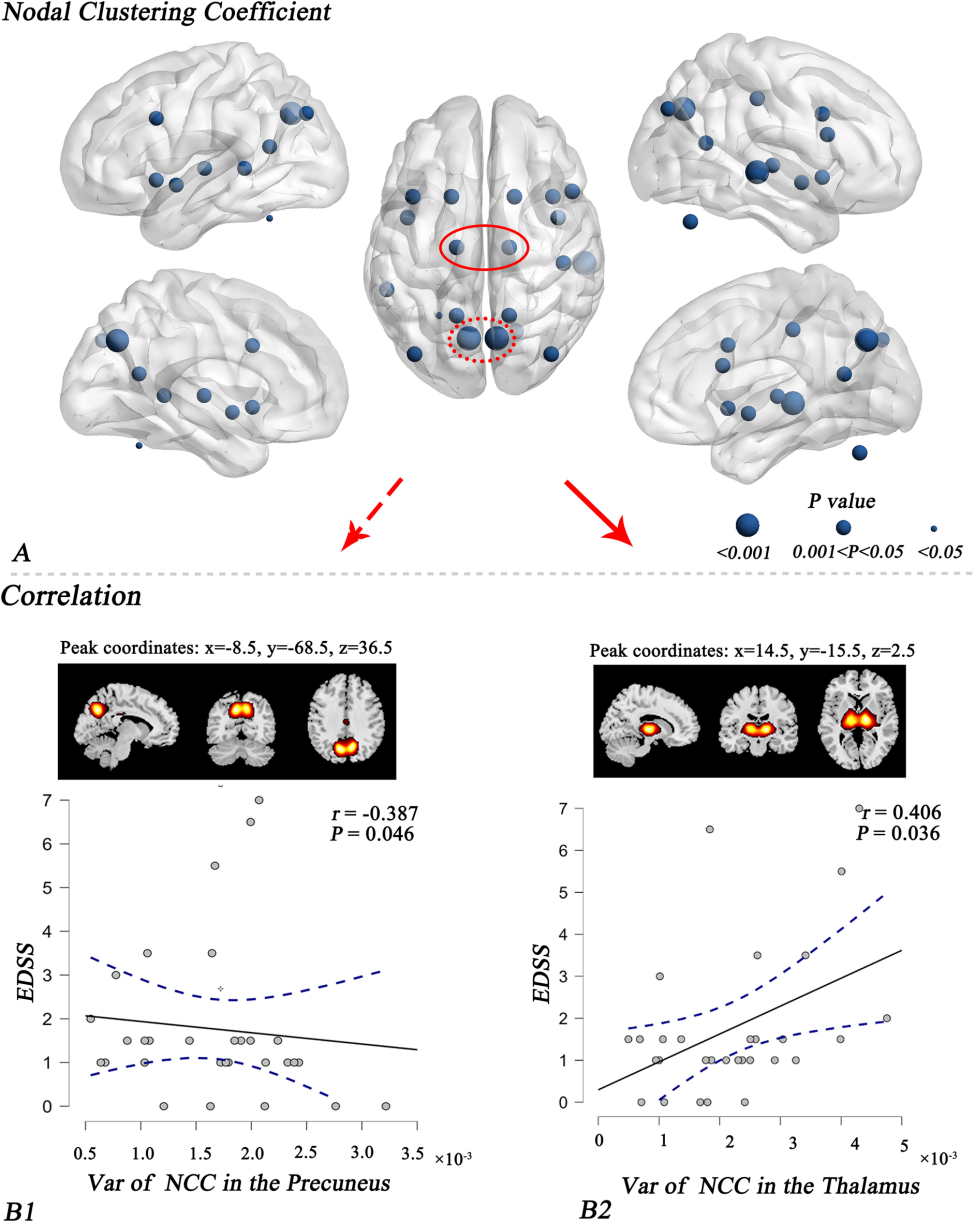
NMOSD patients showed reduced variance for nodal clustering coefficients (**A**) and reduced correlations between EDSS scores and variance for the precuneus (**B1**) and thalamus (**B2**). *Note*: EDSS, Expanded Disability Status Scale; Var, variance; NCC, nodal clustering coefficient; L, left; R, right.

In terms of the nodal properties, patients with NMOSD had lower variance for degree centrality than HC (*P* < 0.05, FDR corrected) (Fig. 4C,D), including IC34 (bilateral anterior cingulate cortex, ACC) and IC35 (bilateral insula). Furthermore, the widespread variance of the nodal clustering coefficients was significantly decreased in NMOSD patients compared to that in HC (*P* < 0.05, FDR corrected), mainly in the DMN, SCN, and CN, such as IC9 (bilateral precuneus) and IC20 (bilateral thalamus) (Fig. 5A). The statistical parameters of all state-related and topological measures of dFNC (including effect size, median and quartile) were shown in Table S4.

The correlation analysis revealed that higher EDSS scores were related to decreased variance for the clustering coefficients of the bilateral precuneus (*r* =  − 0.387, *P* = 0.046), and lower EDSS scores were correlated with decreased variance for the thalamus (*r* = 0.406, *P* = 0.036) in NMOSD patients (Fig. 5B1,B2).

#### Topological properties of sFNC

Graph theory analysis of the sFNC matrix showed no significant differences between patients with NMOSD and HC.

## Discussion

The human brain is a complex, dynamic network with considerable functional interactions. We used a data-driven ICA approach to construct a large-scale functional network and evaluated potential alterations in sFNC and dFNC and their topological properties in NMOSD patients. In the present study, we observed the following results: (1) NMOSD patients showed significant abnormalities in state-related and topological properties associated with dFNC, even when the sFNC did not differ significantly between groups. (2) Specifically, NMOSD patients showed increased occurrence and dwell time in the lowly connected state (State 1) with fewer transitions between states than HC. These alterations were related with more severe disability. (3) Moreover, in terms of the network topology, NMOSD patients displayed altered variability of the global and local topological properties, which were correlated with increased disability.

### Strength and topological properties of sFNC in NMOSD patients

We analyzed alterations in sFNC in NMOSD patients and discovered that the sFNC strength and topological properties could not be used to differentiate NMOSD patients from HC. These findings differ from those reported in some previous studies, which noted extensive functional reorganization of cognitive (e.g., the DMN, SN, and thalamic network) and sensory networks in patients with NMOSD^11,31–33^. In these cohorts, NMOSD patients had a longer disease duration and more severe disability (mean disease duration: 3.6 years; median/mean EDSS score: 4.0/3.5), whereas most of our patients were in the early stages of NMOSD, with a shorter disease duration (mean: 1.8 years), less severe disability (median EDSS score: 1.25) and lesion load (median LV = 1.22 ml). Furthermore, NMOSD patients showed preserved sFNC topological properties compared to HCs, which is consistent with a previous study^22^. Previous research has demonstrated that decreased static functional connectivity may be associated with the white matter lesions^34^. In our study, we did not observe significant differences in strength and topological properties of sFNC, indicating that the static network may remain relatively stable in NMOSD patients with low lesion burden. Additionally, dynamic functional connectivity patterns also could be influenced by lesions. For example, previous study reported that the declined dynamics of functional connectivity within attention networks is closely associated with lesion burden in multiple sclerosis^35^.

### Altered state-related properties of dFNC in NMOSD patients

Three dynamic states were defined for all subjects. State 1 is characterized by overall lower connectivity within and between networks. In terms of temporal dynamics, NMOSD patients showed higher fractional occupancy, increased dwell time in lower connectivity state and fewer transitions were correlated with higher EDSS scores. In other demyelinating diseases, such as multiple sclerosis, most time is spend in a lowly connected state and it may reflect a baseline state in which the brain exhibits minimal neuronal interactions at rest, and the reduced connectivity strengths of a lowly connected state was associated with decreased motor and cognitive performance^36,37^. Our findings suggest that the specific functional state dynamics have clinical relevance and may serve as potential neuroimaging markers for disability assessment in NMOSD. Monitoring the occurrence of lowly connected state and transitions between states could provide valuable information about disease progression and disability severity in individual patients.

### Altered global properties of dFNC in NMOSD patients

For global network analysis, a previous study reported that the small-world parameters did not change in NMOSD patients, which is consistent with our sFNC results^22^. However, NMOSD patients showed a decreased variability for small-worldness and an increased variability for shortest path length. The small-worldness and shortest path length characterize an optimal balance and efficiency between local specialization and global integration during information transmission^18^. The altered global properties suggested the inefficient and unbalanced information processing of functional networks in patients with NMOSD. In addition, previous works found that NMOSD patients exhibited alterations in small-world properties of the structural network, manifested as increased shortest path length and small-worldness, regardless of the network efficiency, which is consistent with the topological dynamics observed in our functional networks^21^.

### Altered nodal properties of dFNC in NMOSD patients

Nodal properties studies in NMOSD have shown that clinical dysfunction is related to an inefficient network, as seen by decreased temporal variability of degree centrality and clustering coefficient in widespread brain regions, mainly involving the DMN, SMN and BG. In particular, reduced temporal variability of the clustering coefficients in the precuneus and thalamus was related to the disability. The precuneus, a part of the higher-order DMN, was mainly associated with cognitive impairment in NMOSD patients. Recently, it was suggested that different patterns of time-varying connectivity in the precuneus might explain cognitive disorders and depression in patients with NMOSD better than static connectivity^15^. The precuneus is also an interface between cognition and action and is involved in many cognitive and motor-related tasks, including motor execution, motor imagery, and coordination between attention and motor function^38,39^. Reduced efficiency and flexibility of the precuneus may thus contribute to the development of neurological dysfunction in patients with NMOSD. Furthermore, the thalamus receives sensory input from the spinothalamic tracts, the central spinal cord region associated with NMOSD lesions, and regulates sensorimotor integration in the cerebral cortex^40^. This supports our results that decreased information efficiency in the thalamus may be associated with disability in NMOSD patients. Structural and functional abnormalities of the thalamus are widely observed in patients with NMOSD and are strongly related to severe fatigue^41^, neuropathic pain^42^ and cognitive impairment^43^.

NMOSD patients also exhibited reduced temporal variability of degree centrality in the insula and ACC. The insula and ACC are the strongest hub regions in the DMN and SN, respectively, playing important roles in integrating information related to emotional regulation and cognitive control^44^. The decreased variability of degree centrality may indicate reduced efficiency and flexibility in information exchanges among core hubs in the cognitive networks of NMOSD patients. These altered topology indicate disruptions within the brain network with efficient information processing and integration, contributing to functional impairments associated with NMOSD. Evaluating these network properties may help to understand the extent of neurological damage and clinical consequences in NMOSD.

This study had some limitations. The sample size of our study was small, and studies with larger sample sizes are needed in the future. Moreover, our study is a retrospective design, the NMOSD patients with mild disability were primarily included. Therefore, future research should investigate potential alterations of sFNC nad dFNC among different subgroups of NMOSD, such as moderate or severe disability. Finally, structural network changes should be investigated to improve our understanding of functional network changes, including time variability, in patients with NMOSD.

## Conclusion

NMOSD patients showed higher fractional occupancy, increased dwell times in the lowly connected state and fewer transitions than HC, as well as decreased degree centrality and clustering coefficients for hub nodes, which were related to physical disability. Future longitudinal studies should investigate whether these temporal and topological properties of dynamic hypoconnectivity can be used as imaging markers for disability and disease progression in NMOSD patients.

### Supplementary Information

Supplementary Information 1.

Supplementary Information 2.

Supplementary Information 3.

Supplementary Information 4.

## Data Availability

Correspondence and requests for the data could be addressed to our corresponding author, Fuqing Zhou.

## References

[1] Collongues N (2014). Characterization of neuromyelitis optica and neuromyelitis optica spectrum disorder patients with a late onset. Mult. Scler. J..

[2] Du Q (2021). Mortality of neuromyelitis optica spectrum disorders in a Chinese population. Ann. Clin. Transl. Neuro..

[3] Cacciaguerra L (2020). Spinal cord atrophy in neuromyelitis optica spectrum disorders is spatially related to cord lesions and disability. Radiology..

[4] Duan Y (2014). White matter atrophy in brain of neuromyelitis optica: A voxel-based morphometry study. Acta Radiol..

[5] Hyun JW (2017). Deep gray matter atrophy in neuromyelitis optica spectrum disorder and multiple sclerosis. Eur. J. Neurol..

[6] Bigaut K (2019). Resting-state functional MRI demonstrates brain network reorganization in neuromyelitis optica spectrum disorder (NMOSD). PLoS ONE.

[7] Han Y (2020). Functional connectivity alterations in neuromyelitis optica spectrum disorder: Correlation with disease duration and cognitive impairment. Clin. Neuroradiol..

[8] Yang Y (2022). Altered functional connectivity associated with cognitive impairment in neuromyelitis optica spectrum disorder. Mult. Scler. Relat. Disord..

[9] Yang L (2022). The role of basal ganglia network in neural plasticity in neuromyelitis optica spectrum disorder with myelitis. Mult. Scler. Relat. Disord..

[10] Savoldi F (2020). Functional brain connectivity abnormalities and cognitive deficits in neuromyelitis optica spectrum disorder. Mult. Scler..

[11] Rocca MA (2019). Cross-modal plasticity among sensory networks in neuromyelitis optica spectrum disorders. Mult. Scler..

[12] Jalilianhasanpour R (2021). Dynamic brain connectivity in resting state functional MR imaging. Neuroimaging Clin. N. Am..

[13] Filippi M, Spinelli EG, Cividini C, Agosta F (2019). Resting state dynamic functional connectivity in neurodegenerative conditions: A review of magnetic resonance imaging findings. Front. Neurosci..

[14] Hutchison, R. M., et al. Dynamic functional connectivity: promise, issues, and interpretations. *Neuroimage.* 360–378 (2013) 10.1016/j.neuroimage.2013.05.079 PMC3807588 23707587

[15] Cacciaguerra L (2022). Time-varying connectivity of the precuneus and its association with cognition and depressive symptoms in neuromyelitis optica: A pilot MRI study. Mult. Scler..

[16] Vecchio F, Miraglia F, Maria Rossini P (2017). Connectome: Graph theory application in functional brain network architecture. Clin. Neurophysiol. Pract..

[17] Sporns O (2011). The human connectome: A complex network. Ann. N. Y. Acad. Sci..

[18] Guye M, Bettus G, Bartolomei F, Cozzone PJ (2010). Graph theoretical analysis of structural and functional connectivity MRI in normal and pathological brain networks. MAGMA.

[19] Li H, Jia X, Li Y, Jia X, Yang Q (2021). Aberrant amplitude of low-frequency fluctuation and degree centrality within the default mode network in patients with vascular mild cognitive impairment. Brain Sci..

[20] Cho EB (2018). White matter network disruption and cognitive dysfunction in neuromyelitis optica spectrum disorder. Front. Neurol..

[21] Zheng Q (2021). Altered structural networks in neuromyelitis optica spectrum disorder related with cognition impairment and clinical features. Mult. Scler. Relat. Disord..

[22] Cho EB (2022). Disrupted structural network of inferomedial temporal regions in relapsing-remitting multiple sclerosis compared with neuromyelitis optica spectrum disorder. Sci. Rep..

[23] Jenkinson M, Bannister P, Brady M, Smith S (2002). Improved optimization for the robust and accurate linear registration and motion correction of brain images. Neuroimage.

[24] Allen EA (2011). A baseline for the multivariate comparison of resting-state networks. Front. Syst. Neurosci..

[25] Yeo BT (2011). The organization of the human cerebral cortex estimated by intrinsic functional connectivity. J. Neurophysiol..

[26] Allen EA (2014). Tracking whole-brain connectivity dynamics in the resting state. Cereb. Cortex.

[27] Zhang J (2011). Disrupted brain connectivity networks in drug-naive, first-episode major depressive disorder. Biol. Psychiatry.

[28] Tu Y (2019). Abnormal thalamocortical network dynamics in migraine. Neurology..

[29] Pang X (2021). Abnormal static and dynamic functional connectivity in left and right temporal lobe epilepsy. Front. Neurosci..

[30] Huang C (2023). Altered dynamic functional network connectivity and topological organization variance in patients with white matter hyperintensities. J. Neurosci. Res..

[31] Zhang Y (2022). Brain structural and functional connectivity alterations are associated with fatigue in neuromyelitis optica spectrum disorder. BMC Neurol..

[32] Chavarro VS (2020). Visual system damage and network maladaptation are associated with cognitive performance in neuromyelitis optica spectrum disorders. Mult. Scler. Relat. Disord..

[33] d'Ambrosio A (2020). Reduced dynamics of functional connectivity and cognitive impairment in multiple sclerosis. Mult. Scler..

[34] Soares JM (2021). Alterations in functional connectivity are associated with white matter lesions and information processing efficiency in multiple sclerosis. Brain Imaging Behav..

[35] Huang M (2019). White matter lesion loads associated with dynamic functional connectivity within attention network in patients with relapsing-remitting multiple sclerosis. J. Clin. Neurosci..

[36] Hidalgo de la Cruz M (2021). Dynamic functional connectivity in the main clinical phenotypes of multiple sclerosis. Brain Connect..

[37] Liu Y (2012). Altered topological organization of white matter structural networks in patients with neuromyelitis optica. PLoS ONE.

[38] Zhang S, Li CS (2012). Functional connectivity mapping of the human precuneus by resting state fMRI. Neuroimage.

[39] Wenderoth N, Debaere F, Sunaert S, Swinnen SP (2005). The role of anterior cingulate cortex and precuneus in the coordination of motor behaviour. Eur. J. Neurosci..

[40] Sherman SM (2016). Thalamus plays a central role in ongoing cortical functioning. Nat. Neurosci..

[41] Seok JM (2022). Association of subcortical structural shapes with fatigue in neuromyelitis optica spectrum disorder. Sci. Rep..

[42] Asseyer S (2020). Ventral posterior nucleus volume is associated with neuropathic pain intensity in neuromyelitis optica spectrum disorders. Mult. Scler. Relat. Disord..

[43] Wang Q (2015). Gray matter volume reduction is associated with cognitive impairment in neuromyelitis optica. AJNR Am. J. Neuroradiol..

[44] Centanni SW, Janes AC, Haggerty DL, Atwood B, Hopf FW (2021). Better living through understanding the insula: Why subregions can make all the difference. Neuropharmacology.

