# Supplementary material for: Temporal and topological properties of dynamic networks reflect disability in patients with neuromyelitis optica spectrum disorders
Source: Sci Rep. 2024 Feb 20;14:4199. doi: 10.1038/s41598-024-54518-7 (PMC10879085; doi:10.1038/s41598-024-54518-7)
Supplement: Supplementary file 1 — Supplementary Information 1. [file 41598_2024_54518_MOESM1_ESM.docx]

Table S1 Brief descriptions of topological metrics examined in our study.

| Topological parameters | | Descriptions |
| --- | --- | --- |
| **Global measures** | | |
| Clustering coefficient (*aCp*) | The *aCp* of a network is the average of the clustering coefficients over all nodes and quantifies the local interconnectivity of a network. | |
| Normalized clustering coefficient (aGamma) | The *aGamma* is calculated as the *aCp* of the real networks divided by the mean *aCp* of 100 random networks. It is a measure used to assess the clustering tendency in a network | |
| Shortest path length (*aLp*) | The *aLp* of a network is the shortest path length required to link one node to another, averaged overall all pairs of nodes. The *aLp* is an indicator of overall routing efficiency of a network. | |
| Normalized characteristic path length (aLambda) | The *aLambda* is calculated as the *aLp* of the real networks divided by the mean *aLp* of 100 random networks. The *aLambda* <1 indicates that the network has a shorter average path length compared to the reference random networks | |
| Small-worldness (aSigma) | The *aSigma* is calculated as *aGamma* divided by *aLambda*. The small-worldness indicating the extent of a network between randomness and order | |
| **Regional metrics** | | |
| Nodal clustering coefficient | Nodal clustering coefficient reflects the local interconnectivity or cliques among the neighbors of a given node. | |
| Degree centrality | The number of connections linked directly to a node. | |
| Nodal efficiency | How efficient an index node communicates with the other nodes. | |
