# Supplementary material for: Temporal and topological properties of dynamic networks reflect disability in patients with neuromyelitis optica spectrum disorders
Source: Sci Rep. 2024 Feb 20;14:4199. doi: 10.1038/s41598-024-54518-7 (PMC10879085; doi:10.1038/s41598-024-54518-7)
Supplement: Supplementary file 2 — Supplementary Information 2. [file 41598_2024_54518_MOESM2_ESM.docx]

| Imaging findings | Frequency, N (%) |
| --- | --- |
| Typical lesions |  |
| Area postrema | 4（13%） |
| Dorsal brainstem | 5（17%） |
| Diencephalon | 3（10%） |
| Adjacent to lateral ventricle | 2（7%） |
| Hemispheric | 1（3%） |
| Non-typical small lesions | 3（10%） |
| No lesion | 20 (67%) |

Table S2 Summary of brain lesions in patients with NMOSD
