# Supplementary material for: Temporal and topological properties of dynamic networks reflect disability in patients with neuromyelitis optica spectrum disorders
Source: Sci Rep. 2024 Feb 20;14:4199. doi: 10.1038/s41598-024-54518-7 (PMC10879085; doi:10.1038/s41598-024-54518-7)
Supplement: Supplementary file 3 — Supplementary Information 3. [file 41598_2024_54518_MOESM3_ESM.docx]

Table S3 the MNI peak coordinate of ICs and RSNs.

| RSNs | Brain Regions | ICs number | MNI peak coordinate | | |
| --- | --- | --- | --- | --- | --- |
|  |  |  | X | Y | Z |
| DMN | Precuneus_B | 9 | -8.5 | -68.5 | 36.5 |
|  | Precuneus_B | 14 | 16.5 | -41.5 | 41.5 |
|  | Posterior Cingulate Gyrus_B | 16 | -3.5 | -29.5 | 30.5 |
|  | Anterior Cingulate Gyrus_B | 28 | -9.5 | 26.5 | -3.5 |
|  | Anterior Cingulate Gyrus_B | 34 | 2.5 | 36.5 | 14.5 |
|  | Angular Gyrus_B | 39 | 29.5 | -59.5 | 44.5 |
|  | Paracingulate Gyrus_B | 43 | 2.5 | 53.5 | 21.5 |
|  | Precuneous_B | 53 | -3.5 | -57.5 | 24.5 |
|  | Precuneus_B | 61 | -14.5 | -54.5 | 15.5 |
|  | Angular Gyrus_B | 66 | -51.5 | -51.5 | 38.5 |
| SMN | Precentral Gyrus_B | 1 | 50.5 | -6.5 | 33.5 |
|  | Postcentral Gyrus_B | 17 | 45.5 | -24.5 | 41.5 |
| AUN | Middle Temporal Gyrus_B | 69 | -54.5 | -39.5 | 2.5 |
|  | Superior Temporal Gyrus_B | 73 | 57.5 | -24.5 | -0.5 |
|  | Superior Temporal Gyrus_B | 74 | -41.5 | -30.5 | 12.5 |
| VIS | Middle Occipital Gyrus_B | 8 | -24.5 | -92.5 | -0.5 |
|  | Calcarine_B | 6 | 8.5 | -87.5 | 6.5 |
|  | Lingual Gyrus_B | 27 | -15.5 | -74.5 | -6.5 |
|  | Cuneus_B | 30 | 9.5 | -83.5 | 29.5 |
|  | Middle Temporal Gyrus_B | 32 | 50.5 | -65.5 | 5.5 |
|  | Occipital Middle Gyrus_B | 33 | 38.5 | -77.5 | 35.5 |
|  | Inferior Occipital Gyrus_B | 46 | 40.5 | -72.5 | -0.5 |
|  | Calcarine_L | 49 | -15.5 | -65.5 | 8.5 |
| ATN | Insula_B | 2 | -42.5 | 0.5 | -6.5 |
|  | Insula_B | 21 | -29.5 | 26.5 | 0.5 |
|  | Precentral Gyrus_L | 26 | -48.5 | 5.5 | 23.5 |
|  | Rolandic Operculum_B | 35 | 44.5 | 3.5 | 9.5 |
|  | Middle Frontal Gyrus_B | 45 | -35.9 | 9.5 | 36.5 |
|  | Superior Temporal Gyrus_R | 52 | 44.5 | -42.5 | 15.5 |
|  | Supramarginal Gyrus_B | 67 | -57.5 | -33.5 | 30.5 |
| PFN | Superior Frontal Gyrus_B | 18 | 15.5 | 27.5 | 38.5 |
|  | Middle Frontal Gyrus_B | 22 | -29.5 | 50.5 | 9.5 |
|  | Angular Gyrus_B | 36 | 45.5 | -59.5 | 45.5 |
|  | Middle Frontal Gyrus_B | 38 | -39.5 | 12.5 | 32.5 |
|  | Inferior Frontal Gyrus_B | 57 | -41.5 | 42.5 | 2.5 |
|  | Inferior Frontal Gyrus_L | 62 | 50.5 | 15.5 | 20.5 |
| SCN | Thalamus_B | 20 | 14.5 | -15.5 | 2.5 |
|  | Putamen_B | 23 | 27.5 | 5.5 | 0.5 |
|  | Caudate_B | 25 | -17.5 | 12.5 | -3.5 |
| CN | Vermis 4 5 | 5 | 2.5 | -56.5 | -14.5 |
|  | Cerebelum Crus1_B | 13 | -21.5 | -74.5 | -30.5 |
|  | Cerebelum 6_R | 54 | 18.5 | -63.5 | -29.5 |
|  | Cerebelum 6_L | 55 | -24.5 | -54.5 | -26.5 |

RSNs, resting-state networks; DMN, default mode network; SMN, sensorimotor network; ADN, auditory network; VIS, visual network; ATN, attention network; FPN, frontoparietal network; SCN, subcortical network; CN, cerebellar network. ICs, independent components; B, bilateral; R, right; L, left.
