# Supplementary material for: Temporal and topological properties of dynamic networks reflect disability in patients with neuromyelitis optica spectrum disorders
Source: Sci Rep. 2024 Feb 20;14:4199. doi: 10.1038/s41598-024-54518-7 (PMC10879085; doi:10.1038/s41598-024-54518-7)
Supplement: Supplementary file 4 — Supplementary Information 4. [file 41598_2024_54518_MOESM4_ESM.docx]

Table S4 Summary of statistical parameters in our study.

|  | NMOSD: Median (quartile) | HC: Median (quartile) | Uncorrected P values | Effect size |
| --- | --- | --- | --- | --- |
| **State-related properties** |  |  |  |  |
| Fraction time of State 1 | 0.823 (0.549-0.993) | 0.00 (0.00-0.00) | **<0.001** | 0.993 |
| Fraction time of State 2 | 0.328 (0.000-0.328) | 0.99 (0.68-1.00) | **<0.001** | 0.833 |
| Fraction time of State 3 | 0.00 (0.00-0.00) | 0.00 (0.00-0.037) | 0.070 | 0.158 |
| Mean dwell time of State 1 | 51.833 (22.750-179.50) | 0.00 (0.00-0.00) | **<0.001** | 0.981 |
| Mean dwell time of State 2 | 11.25 (0.000-18.00) | 98.50 (47.00-198.00) | **<0.001** | 0.826 |
| Mean dwell time of State 3 | 0.00 (0.00-0.00) | 0.00 (0.00-12.00) | 0.090 | 0.153 |
| Number of transitions | 5.50 (0.50-7.00) | 1.00 (0.00-3.00) | **0.028** | 0.289 |
| **Global metrics** |  |  |  |  |
| Variance of aCp | 0.00037 (0.00023-0.00046) | 0.00032 (0.00026-0.00045) | 0.671 | 0.059 |
| Variance of aGamma | 0.005 (0.003-0.007) | 0.006 (0.004-0.010) | 0.106 | 0.222 |
| Variance of aLp | 0.009 (0.003-0.019) | 0.003 (0.002-0.009) | **0.005** | 0.379 |
| Variance of aLambda | 0.000057 (0.000024-0.000057) | 0.000032 (0.000017-0.000066) | 0.179 | 0.185 |
| Variance of aSigma | 0.004 (0.003-0.005) | 0.005 (0.003-0.008) | **0.034** | 0.292 |
| **Nodal metrics** |  |  |  |  |
| **Variance of nodal clustering coefficient for ICs** | | | | |
| Precuneus_B (IC9) | 0.002(0.001-0.002) | 0.002(0.002-0.003) | **< .001** | 0.468 |
| Precuneus_B (IC14) | 0.002(0.001-0.003) | 0.003(0.002-0.004) | 0.022 | 0.313 |
| Posterior Cingulate Gyrus_B (IC16) | 0.002(0.001-0.003) | 0.003(0.002-0.003) | 0.009 | 0.357 |
| Anterior Cingulate Gyrus_B (IC28) | 0.002(0.001-0.002) | 0.003(0.002-0.003) | 0.016 | 0.327 |
| Anterior Cingulate Gyrus_B (IC34) | 0.002(0.001-0.002) | 0.002(0.002-0.003) | 0.063 | 0.255 |
| Angular Gyrus_B (IC39) | 0.001(0.001-0.002) | 0.002(0.001-0.003) | 0.225 | 0.167 |
| Paracingulate Gyrus_B (IC43) | 0.002(0.001-0.002) | 0.002(0.001-0.002) | 0.961 | 0.007 |
| Precuneous B (IC53) | 0.001(0.001-0.002) | 0.001(0.001-0.002) | 0.324 | 0.136 |
| Precuneus_B (IC61) | 0.001(0.001-0.002) | 0.002(0.001-0.003) | **0.002** | 0.428 |
| Angular Gyrus_B (IC66) | 0.001(0.001-0.002) | 0.002(0.001-0.003) | 0.055 | 0.264 |
| Precentral Gyrus_B (IC1) | 0.002(0.001-0.003) | 0.003(0.002-0.003) | 0.014 | 0.335 |
| Postcentral Gyrus_B (IC17) | 0.001(0.001-0.002) | 0.002(0.001-0.003) | 0.009 | 0.354 |
| Middle Temporal Gyrus_B (IC69) | 0.002(0.001-0.002) | 0.002(0.002-0.003) | 0.008 | 0.36 |
| Superior Temporal Gyrus_B (IC73) | 0.002(0.001-0.002) | 0.002(0.002-0.003) | **< .001** | 0.52 |
| Superior Temporal Gyrus_B (IC74) | 0.001(0.001-0.002) | 0.002(0.001-0.003) | 0.022 | 0.314 |
| Middle Occipital Gyrus_B (IC8) | 0.002(0.001-0.002) | 0.002(0.002-0.003) | 0.011 | 0.345 |
| Calcarine_B (IC6) | 0.002(0.001-0.002) | 0.002(0.001-0.003) | 0.648 | 0.064 |
| Lingual Gyrus_B (IC27) | 0.001(0.001-0.002) | 0.001(0.001-0.002) | 0.58 | 0.077 |
| Cuneus_B (IC30) | 0.001(0.001-0.002) | 0.001(0.001-0.002) | 0.834 | 0.03 |
| Middle Temporal Gyrus_B (IC32) | 0.002(0.001-0.002) | 0.002(0.001-0.002) | 0.488 | 0.096 |
| Occipital Middle Gyrus_B (IC33) | 0.002(0.001-0.002) | 0.002(0.002-0.003) | 0.004 | 0.391 |
| Inferior Occipital Gyrus_B (IC46) | 0.002(0.001-0.002) | 0.002(0.001-0.003) | 0.018 | 0.323 |
| Calcarine_L (IC49) | 0.001(0.001-0.002) | 0.002(0.001-0.003) | 0.009 | 0.354 |
| Insula_B (IC2) | 0.003(0.002-0.003) | 0.003(0.002-0.005) | 0.015 | 0.332 |
| Insula_B (IC21) | 0.002(0.001-0.003) | 0.003(0.002-0.003) | 0.005 | 0.385 |
| Precentral Gyrus_L (IC26) | 0.002(0.002-0.003) | 0.002(0.002-0.003) | 0.251 | 0.159 |
| Insula_B (IC35) | 0.002(0.001-0.002) | 0.002(0.001-0.003) | 0.587 | 0.076 |
| Middle Frontal Gyrus_B (IC45) | 0.001(0.001-0.002) | 0.002(0.001-0.002) | 0.015 | 0.332 |
| Superior Temporal Gyrus_R (IC52) | 0.002(0.001-0.002) | 0.002(0.002-0.003) | 0.033 | 0.292 |
| Supramarginal Gyrus_B (IC67) | 0.001(0.001-0.002) | 0.001(0.001-0.002) | 0.625 | 0.068 |
| Superior Frontal Gyrus_B (IC18) | 0.002(0.001-0.002) | 0.002(0.002-0.004) | 0.019 | 0.32 |
| Middle Frontal Gyrus_B (IC22) | 0.001(0.001-0.003) | 0.003(0.002-0.003) | 0.005 | 0.379 |
| Angular Gyrus_B (IC36) | 0.001(0.001-0.002) | 0.002(0.001-0.002) | 0.099 | 0.227 |
| Middle Frontal Gyrus_B (IC38) | 0.001(0.001-0.002) | 0.002(0.001-0.003) | **< .001** | 0.453 |
| Inferior Frontal Gyrus_B (IC57) | 0.002(0.001-0.002) | 0.002(0.002-0.003) | 0.104 | 0.224 |
| Inferior Frontal Gyrus_L (IC62) | 0.001(0.001-0.002) | 0.002(0.002-0.003) | **< .001** | 0.444 |
| Thalamus_B (IC20) | 0.002(0.001-0.003) | 0.003(0.002-0.004) | 0.006 | 0.372 |
| Putamen_B (IC23) | 0.002(0.002-0.003) | 0.003(0.002-0.004) | 0.031 | 0.295 |
| Caudate_B (IC25) | 0.002(0.002-0.003) | 0.004(0.002-0.004) | 0.011 | 0.347 |
| Vermis 4 5 (IC5) | 0.002(0.002-0.003) | 0.003(0.002-0.004) | **0.002** | 0.425 |
| Cerebelum Crus1 B (IC13) | 0.001(0.001-0.002) | 0.002(0.001-0.003) | 0.01 | 0.351 |
| Cerebelum 6_R (IC54) | 0.003(0.002-0.003) | 0.003(0.002-0.004) | 0.006 | 0.373 |
| Cerebelum 6_L (IC55) | 0.002(0.002-0.003) | 0.003(0.002-0.004) | 0.008 | 0.361 |
| **Variance of degree centrality for ICs** | | | | |
| Precuneus_B (IC9) | 1.380(1.023-1.943) | 1.788(1.408-2.607) | 0.012 | 0.342 |
| Precuneus_B (IC14) | 1.411(1.071-1.800) | 1.785(1.080-2.654) | 0.153 | 0.197 |
| Posterior Cingulate Gyrus_B (IC16) | 1.488(1.060-2.004) | 1.683(1.172-2.393) | 0.602 | 0.073 |
| Anterior Cingulate Gyrus_B (IC28) | 1.696(0.984-2.017) | 1.578(1.170-2.587) | 0.551 | 0.083 |
| Anterior Cingulate Gyrus_B (IC34) | 1.443(0.786-2.058) | 1.772(1.284-2.726) | **0.003** | 0.397 |
| Angular Gyrus_B (IC39) | 1.534(1.057-2.481) | 1.665(1.157-2.548) | 0.551 | 0.083 |
| Paracingulate Gyrus_B (IC43) | 1.288(0.956-1.986) | 2.050(1.211-2.784) | 0.012 | 0.342 |
| Precuneous B (IC53) | 1.239(0.948-1.759) | 1.844(1.293-2.421) | 0.017 | 0.326 |
| Precuneus_B (IC61) | 1.639(1.086-2.495) | 1.921(1.241-2.519) | 0.335 | 0.133 |
| Angular Gyrus_B (IC66) | 1.611(1.319-1.810) | 2.017(1.332-2.759) | 0.031 | 0.295 |
| Precentral Gyrus_B (IC1) | 1.668(0.969-2.147) | 2.152(1.184-2.647) | 0.021 | 0.316 |
| Postcentral Gyrus_B (IC17) | 1.434(1.085-2.091) | 1.979(1.334-2.624) | 0.041 | 0.28 |
| Middle Temporal Gyrus_B (IC69) | 1.469(1.021-2.474) | 2.055(1.428-2.885) | 0.031 | 0.295 |
| Superior Temporal Gyrus_B (IC73) | 1.455(1.047-1.935) | 1.933(1.295-2.595) | 0.077 | 0.243 |
| Superior Temporal Gyrus_B (IC74) | 1.269(0.878-1.942) | 1.914(1.176-2.833) | 0.008 | 0.361 |
| Middle Occipital Gyrus_B (IC8) | 1.667(1.188-2.268) | 1.881(1.314-2.506) | 0.363 | 0.126 |
| Calcarine_B (IC6) | 1.273(1.088-1.974) | 1.870(1.254-2.534) | 0.095 | 0.23 |
| Lingual Gyrus_B (IC27) | 1.165(0.945-2.020) | 1.644(1.312-2.096) | 0.067 | 0.252 |
| Cuneus_B (IC30) | 1.390(1.098-1.797) | 1.531(1.196-2.061) | 0.558 | 0.081 |
| Middle Temporal Gyrus_B (IC32) | 1.755(1.194-2.573) | 1.787(1.279-2.314) | 0.893 | 0.019 |
| Occipital Middle Gyrus_B (IC33) | 1.825(1.212-2.355) | 1.633(1.231-2.556) | 0.792 | 0.037 |
| Inferior Occipital Gyrus_B (IC46) | 1.442(0.897-2.132) | 1.920(1.320-2.796) | 0.047 | 0.273 |
| Calcarine_L (IC49) | 1.248(0.900-1.832) | 2.033(1.350-2.694) | 0.008 | 0.36 |
| Insula_B (IC2) | 1.268(0.728-1.846) | 1.792(1.048-2.651) | 0.046 | 0.274 |
| Insula_B (IC21) | 1.420(1.160-1.918) | 2.021(1.526-2.879) | 0.004 | 0.393 |
| Precentral Gyrus_L (IC26) | 1.410(1.150-1.917) | 1.851(1.377-2.236) | 0.026 | 0.305 |
| Insula_B (IC35) | 1.206(1.003-1.777) | 1.956(1.386-2.745) | **0.002** | 0.424 |
| Middle Frontal Gyrus_B (IC45) | 1.390(1.082-1.800) | 1.883(1.307-2.490) | 0.014 | 0.336 |
| Superior Temporal Gyrus_R (IC52) | 1.659(1.257-2.306) | 1.702(1.111-2.348) | 0.97 | 0.006 |
| Supramarginal Gyrus_B (IC67) | 1.346(1.111-1.800) | 1.750(1.152-2.143) | 0.162 | 0.193 |
| Superior Frontal Gyrus_B (IC18) | 1.439(0.979-2.232) | 1.796(1.124-2.668) | 0.357 | 0.127 |
| Middle Frontal Gyrus_B (IC22) | 1.560(1.014-2.044) | 1.796(1.311-2.490) | 0.124 | 0.212 |
| Angular Gyrus_B (IC36) | 1.323(0.854-1.963) | 1.750(1.226-2.493) | 0.012 | 0.344 |
| Middle Frontal Gyrus_B (IC38) | 1.611(1.064-2.046) | 1.824(1.295-2.689) | 0.051 | 0.268 |
| Inferior Frontal Gyrus_B (IC57) | 1.413(1.045-2.174) | 1.894(1.118-2.294) | 0.352 | 0.129 |
| Inferior Frontal Gyrus_L (IC62) | 1.565(1.104-2.140) | 2.000(1.588-2.411) | 0.039 | 0.283 |
| Thalamus_B (IC20) | 1.052(0.648-1.510) | 1.429(1.212-2.164) | 0.011 | 0.348 |
| Putamen_B (IC23) | 1.003(0.746-1.550) | 1.622(1.052-3.164) | 0.006 | 0.376 |
| Caudate_B (IC25) | 1.609(0.995-2.187) | 1.542(1.040-2.242) | 0.735 | 0.047 |
| Vermis 4 5 (IC5) | 1.481(0.941-2.077) | 1.737(1.097-2.579) | 0.217 | 0.17 |
| Cerebelum Crus1 B (IC13) | 1.517(1.004-2.066) | 1.938(1.404-2.721) | 0.018 | 0.323 |
| Cerebelum 6_R (IC54) | 1.254(0.839-1.890) | 1.547(1.180-2.088) | 0.172 | 0.188 |
| Cerebelum 6_L (IC55) | 1.764(1.095-2.175) | 1.556(1.158-2.531) | 0.495 | 0.095 |
| **Variance of nodal efficiency for ICs** | | | | |
| Precuneus_B (IC9) | 0.00057(0.00050-0.00077) | 0.00070(0.00049-0.00089) | 0.289 | 0.147 |
| Precuneus_B (IC14) | 0.00078(0.00048-0.00100) | 0.00072(0.00047-0.00100) | 0.751 | 0.044 |
| Posterior Cingulate Gyrus_B (IC16) | 0.00079(0.00061-0.00100) | 0.00070(0.00052-0.00100) | 0.602 | 0.073 |
| Anterior Cingulate Gyrus_B (IC28) | 0.00066(0.00058-0.00100) | 0.00062(0.00048-0.00100) | 0.655 | 0.062 |
| Anterior Cingulate Gyrus_B (IC34) | 0.00062(0.00040-0.00100) | 0.00070(0.00058-0.00100) | 0.314 | 0.139 |
| Angular Gyrus_B (IC39) | 0.00064(0.00045-0.00088) | 0.00054(0.00038-0.00089) | 0.392 | 0.119 |
| Paracingulate Gyrus_B (IC43) | 0.00056(0.00039-0.00073) | 0.00056(0.00040-0.00081) | 0.61 | 0.071 |
| Precuneous B (IC53) | 0.00052(0.00034-0.00064) | 0.00054(0.00040-0.00069) | 0.495 | 0.095 |
| Precuneus_B (IC61) | 0.00062(0.00046-0.00091) | 0.00066(0.00049-0.00095) | 0.784 | 0.039 |
| Angular Gyrus_B (IC66) | 0.00068(0.00050-0.00088) | 0.00064(0.00044-0.00089) | 0.632 | 0.067 |
| Precentral Gyrus_B (IC1) | 0.00067(0.00046-0.00099) | 0.00080(0.00057-0.00100) | 0.251 | 0.159 |
| Postcentral Gyrus_B (IC17) | 0.00063(0.00046-0.00076) | 0.00070(0.00048-0.00098) | 0.417 | 0.113 |
| Middle Temporal Gyrus_B (IC69) | 0.00066(0.00057-0.00081) | 0.00066(0.00050-0.00098) | 0.551 | 0.083 |
| Superior Temporal Gyrus_B (IC73) | 0.00071(0.00044-0.00088) | 0.00068(0.00052-0.00100) | 0.294 | 0.145 |
| Superior Temporal Gyrus_B (IC74) | 0.00067(0.00041-0.00078) | 0.00069(0.00047-0.00093) | 0.304 | 0.142 |
| Middle Occipital Gyrus_B (IC8) | 0.00074(0.00056-0.00090) | 0.00070(0.00051-0.00091) | 0.876 | 0.022 |
| Calcarine_B (IC6) | 0.00062(0.00045-0.00082) | 0.00058(0.00038-0.00080) | 0.792 | 0.037 |
| Lingual Gyrus_B (IC27) | 0.00056(0.00043-0.00072) | 0.00055(0.00032-0.00079) | 0.784 | 0.039 |
| Cuneus_B (IC30) | 0.00053(0.00043-0.00075) | 0.00043(0.00033-0.00061) | 0.089 | 0.234 |
| Middle Temporal Gyrus_B (IC32) | 0.00082(0.00052-0.00100) | 0.00055(0.00042-0.00095) | 0.055 | 0.264 |
| Occipital Middle Gyrus_B (IC33) | 0.00069(0.00055-0.00100) | 0.00065(0.00049-0.00084) | 0.213 | 0.172 |
| Inferior Occipital Gyrus_B (IC46) | 0.00068(0.00044-0.00094) | 0.00066(0.00053-0.00100) | 0.324 | 0.136 |
| Calcarine_L (IC49) | 0.00053(0.00040-0.00076) | 0.00066(0.00044-0.00082) | 0.289 | 0.147 |
| Insula_B (IC2) | 0.00068(0.00058-0.00100) | 0.00084(0.00063-0.00100) | 0.121 | 0.213 |
| Insula_B (IC21) | 0.00078(0.00057-0.00100) | 0.00079(0.00060-0.00098) | 0.663 | 0.061 |
| Precentral Gyrus_L (IC26) | 0.00068(0.00049-0.00099) | 0.00071(0.00049-0.00093) | 0.961 | 0.007 |
| Insula_B (IC35) | 0.00056(0.00042-0.00073) | 0.00061(0.00040-0.00085) | 0.341 | 0.132 |
| Middle Frontal Gyrus_B (IC45) | 0.00052(0.00040-0.00077) | 0.00064(0.00043-0.00088) | 0.398 | 0.117 |
| Superior Temporal Gyrus_R (IC52) | 0.00071(0.00049-0.00100) | 0.00059(0.00042-0.00081) | 0.089 | 0.234 |
| Supramarginal Gyrus_B (IC67) | 0.00053(0.00036-0.00071) | 0.00048(0.00032-0.00073) | 0.363 | 0.126 |
| Superior Frontal Gyrus_B (IC18) | 0.00074(0.00052-0.00041) | 0.00065(0.00048-0.00091) | 0.41 | 0.114 |
| Middle Frontal Gyrus_B (IC22) | 0.00063(0.00040-0.00095) | 0.00066(0.00055-0.00087) | 0.435 | 0.108 |
| Angular Gyrus_B (IC36) | 0.00058(0.00043-0.00074) | 0.00062(0.00044-0.00080) | 0.335 | 0.133 |
| Middle Frontal Gyrus_B (IC38) | 0.00061(0.00049-0.00071) | 0.00070(0.00043-0.00091) | 0.129 | 0.209 |
| Inferior Frontal Gyrus_B (IC57) | 0.00065(0.00048-0.00097) | 0.00062(0.00050-0.00081) | 0.565 | 0.08 |
| Inferior Frontal Gyrus_L (IC62) | 0.00065(0.00048-0.00100) | 0.00069(0.00045-0.00093) | 0.893 | 0.019 |
| Thalamus_B (IC20) | 0.00068(0.00040-0.00089) | 0.00068(0.00044-0.00100) | 0.186 | 0.182 |
| Putamen_B (IC23) | 0.00073(0.00060-0.00097) | 0.00073(0.00059-0.00100) | 0.776 | 0.04 |
| Caudate_B (IC25) | 0.00077(0.00062-0.00100) | 0.00084(0.00060-0.00100) | 0.868 | 0.024 |
| Vermis 4 5 (IC5) | 0.00085(0.00055-0.00097) | 0.00075(0.00051-0.00100) | 0.851 | 0.027 |
| Cerebelum Crus1 B (IC13) | 0.00064(0.00047-0.00080) | 0.00065(0.00050-0.00090) | 0.474 | 0.099 |
| Cerebelum 6_R (IC54) | 0.00091(0.00066-0.00100) | 0.00077(0.00058-0.00100) | 0.842 | 0.028 |
| Cerebelum 6_L (IC55) | 0.00080(0.00058-0.00099) | 0.00078(0.00057-0.00100) | 0.834 | 0.03 |

Note: The effect size is calculated using the rank-biserial correlation coefficient and is presented as the absolute value, which located 0 to 1. When the effect size is close to 1, it means more significant difference between the two groups.
